# X-ray fluorescence analysis of three late medieval silver chalices associated with Ireland

**DOI:** 10.1186/s40494-024-01240-2

**Published:** 2024-04-25

**Authors:** Veronica Biolcati, Richard Keyes McDonnell, Anna Grace Hoffman, Pádraig Ó Macháin, Małgorzata Krasnodębska-D’Aughton, Daniela Iacopino

**Affiliations:** 1 Tyndall National Institute, Lee Maltings Complex, Dyke Parade, Cork, T12R5CP Ireland; 2 School of History, University College Cork, College Road, Cork, T12K8AF Ireland; 3 Modern Irish Department, University College Cork, College Road, Cork, T12K8AF Ireland

**Keywords:** Metal analysis, Fire gilding, Glass, Enamel, Historical silverware

## Abstract

This paper presents the results of the first X-ray fluorescence (XRF) investigation conducted on three late medieval chalices associated with Ireland: the Ó Learghusa chalice, auctioned as medieval Irish in 2021, does not have a confirmed provenance; the de Burgo-O'Malley chalice, dated 1494, and the TP-IEP chalice, dated 1589, both of Irish provenance. This study effectively addressed the knowledge gap concerning Irish medieval silver chalices composition. The analysis revealed that both the Ó Learghusa and de Burgo-O'Malley chalices were crafted from a silver-copper alloy and adorned using a fire-gilding technique. The blue and green enamels on the de Burgo-O'Malley chalice were found to be constituted by cobalt and iron/copper glasses, respectively. In contrast, the TP-IEP chalice exhibited a more intricate structure, being a composite object with partial silver gilt and with the bowl and base possibly made of a ternary silver-copper-gold alloy. The TP-IEP chalice’s knop displayed glass, simulating gems with transparent, blue, and purple colorations. XRF analysis allowed identification of lead-potash glass, while the red glass displayed a rich iron content and was identified as soda-lime glass. The analysis allowed concluding that the de Burgo-O'Malley chalice had retained its original condition, including its original gilding and enamels, while the Ó Learghusa and TP-IEP chalices appeared to have undergone refurbishment. These significant discoveries contribute to a deeper understanding of the historical context and artistic craftsmanship behind these late medieval chalices, shedding light on their unique stories within Irish art and history.

**Supplementary Information:**

The online version contains supplementary material available at 10.1186/s40494-024-01240-2.

## Introduction

Chalices played a key role in medieval liturgy and were requisites for the celebration of the Eucharist, as they contained consecrated wine, which together with the host, represented the real presence of Christ’s blood and body on the altar [1]. Early Christian chalices were made of various materials, including glass and bronze. However, by the late eighth century, the western Church stipulated that sacred liturgical objects be made of gold (Au) and silver (Ag), because Christ’s blood and body should only be contained within objects created of the most precious materials [2]. These liturgical objects were often composed of a copper–silver (Cu-Ag) alloy as base metal. Cu was added to Ag for increasing the strength and wear resistance of the alloy [3]. For aesthetic reasons, and to add preciousness, the chalices’ metal surface was overlaid with an Au layer, a process called gilding. Among various procedures, during the Middle Ages fire-gilding was the most popular technique. It consisted in the application of a paste of Au powder in liquid mercury (Hg), followed by firing of the artifact at high temperature to induce the evaporation of Hg, which left a solid layer of Au behind [4, 5]. As the choice of chalice materials changed over the centuries—from glass to precious metals—so did the shape of these objects. Early medieval chalices with a wide bowl, a short stem and a circular foot, where substituted in the thirteenth century by chalices in a Gothic style with a narrower bowl, a slender stem, a bulbous knop and a polygonal foot, usually hexagonal or octagonal [6].

XRF has been extensively applied to the characterization and investigation of historical metal alloys, whereby it has been shown of key importance to support hypotheses regarding manufacturing techniques, contemporaneity of the production of various components within the object, later additions, and conservation interventions [7–11]. Advantages of XRF include speed of analysis, no requirements for sample preparation, non-invasiveness, capability to return a precise elemental composition with an approximation of the relative concentrations of constituent elements, also in traces [12, 13]. It’s worth noting that metals and metal alloys primarily yield a signal from the few superficial microns of material. Therefore, in metal studies, this technique can be considered a surface technique [14, 15].

To date, no chalice of proven Irish provenance and date has been subjected to XRF analysis or any other scientific analysis. As a result, no data regarding the elemental composition of liturgical artifacts produced in late medieval Ireland is available. The absence of such information severely limits the understanding of the materials and techniques employed in crafting chalices during that historical period in Ireland. The primary objective of the presented work was to fill this gap by the investigation of three chalices performed through a complementary approach which combined stylistic and historical information with an XRF study of materials and manufacturing techniques. Therefore, following historical analysis, the composition of the chalices metal alloy, the gilding layer, and the enamels and glass (where present), were investigated by XRF. The qualitative examination of XRF data lead to highly valuable insights into the choice and use of materials.

## Research aim

This paper aims to fill a significant gap in our understanding of Irish medieval silver chalices composition, by conducting an insightful X-ray fluorescence (XRF) investigation on three late medieval chalices associated with Ireland: the Ó Learghusa chalice, auctioned as medieval Irish in 2021; the de Burgo-O'Malley chalice, dated 1494; and the TP-IEP chalice, dated 1589. Through elemental analysis, the paper seeks to unravel the intricate material composition and craftsmanship techniques utilized in the creation of these chalices. By delving into these aspects, this research has shed new light on a previously unexplored area, enriching our comprehension of historical metalworking practices in Ireland.

## Methods

Optical microscopy was performed to support visual analysis. A Zeiss Stemi 508 (Carl Zeiss MicroscopyGmbH, Jena, Germany) stereo-microscope, equipped with an Axiocam 208 digital camera with 8 Megapixel resolution, was used to acquire digital images at various magnifications, from 12.5 × to 100 × . The instrument was equipped with visible LED ring illuminator lights.

X-ray fluorescence spectroscopy was performed with a portable, energy dispersive XRF ELIO (XGLab, srl). The spectrometer was equipped with a Peltier-cooled Silicon Drift Detector with a resolution of 135 eV at the manganese (Mn) Kα line (5.9 keV). The excitation source was a low-power (4W, 50 kV) transmission X-ray tube with a Rh anode. Measurements were performed with no filter under an air atmosphere at 50 kV voltage with a tube current of 10 µA. The X-ray generator was coupled with a collimator, allowing a ~ 1 mm-diameter focused spot size on the surface of the metal. The distance of the instrument head from the analyzed areas was ~ 1 cm, and the acquisition time was 90 s for all spectra. For each point of analysis various measurements were taken (10 for the Ó Learghusa chalice, 3 for the de Burgo-O’Malley chalice, and 3 for the TP-IEP chalice). This approach was chosen to average the spectra of each area to obtain a more accurate estimation of constituent elements. Due to the close resemblance of all spectra obtained from the same area, in this paper a representative spectrum is presented for each case. XRF analysis was conducted non-invasively, without any superficial treatment of the objects.

## Materials, results and discussion

### Comparative stylistic analysis of analyzed chalices

**Fig. 1:**
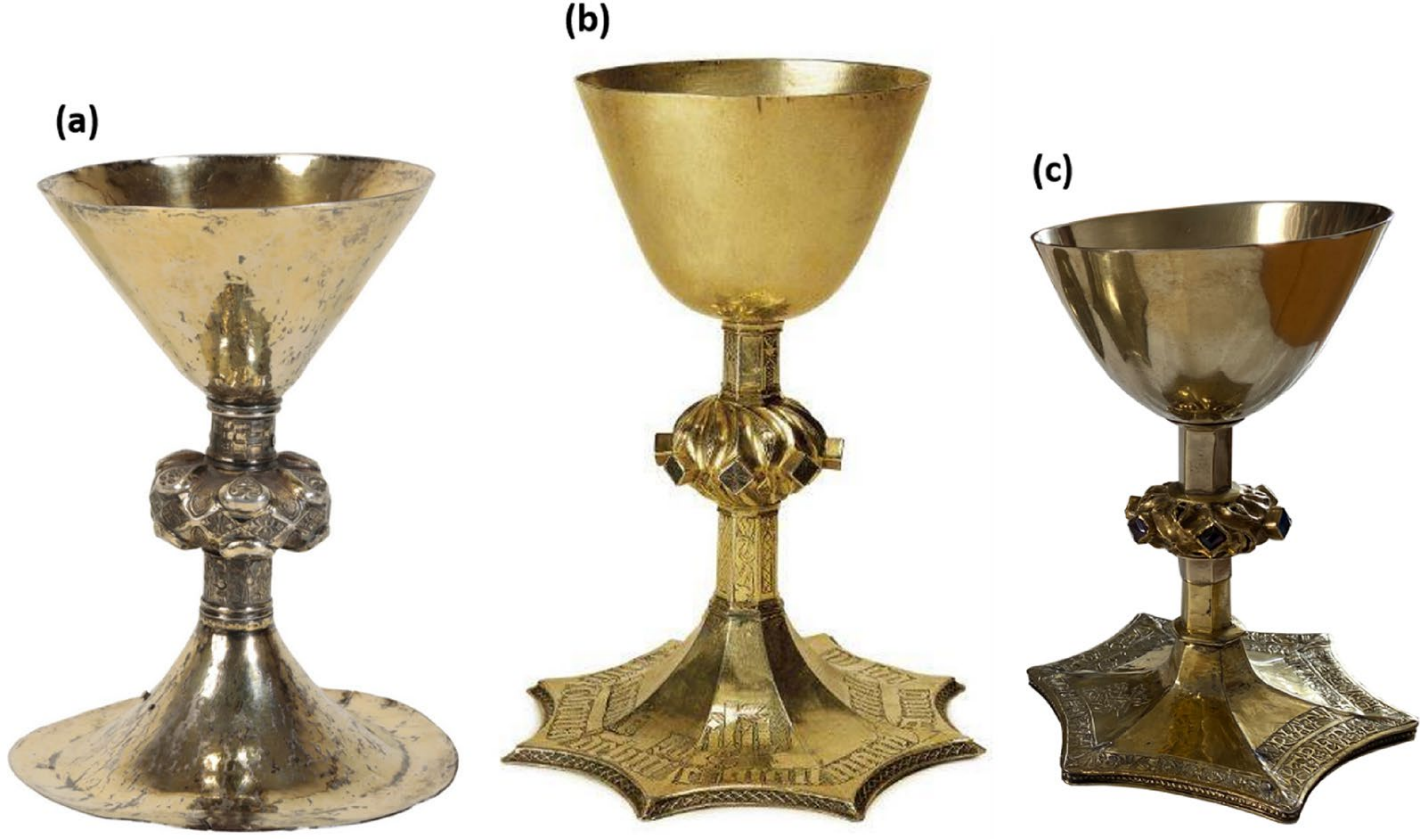
Photographs of analyzed chalices: **a** Ó Learghusa; **b** de Burgo-O’Malley; **c** TP-IEP

Figure 1 shows photographs of the three chalices analyzed in this work. The Ó Learghusa chalice (1a), named as such for the purpose of this paper to reflect the family connection of its last owner. This chalice, auctioned in 2021 by Duke’s Auctioneers in Dorchester, was described in the auction catalog as “an exceptionally rare Irish silver gilt chalice, circa 1480”. The absence of any inscriptions or hallmarks posed a challenge in assigning a specific provenance or date. The de Burgo-O’Malley chalice (1b), is a gilded chalice with enamels, securely dated to the late fifteenth century. It was probably produced in Galway for the Dominican priory at Burrishoole, Co. Mayo [16]. The inscription at the base identified Thomas de Burgo and Grainne O’Malley as its donors and provides the date of 1494 [17]. The third chalice analyzed was the Irish TP-IEP chalice dated by the inscription to 1589 (1c). It was a gilded chalice embellished with colored glass on the knop. Its donors may be identified as Thomas Purcell and his wife Joanna Fitzpatrick [18]. One facet of the foot beard the image of the Paschal Lamb with a cross [17].

The Ó Learghusa, the de Burgo, and the TP-IEP chalices have been chosen for this study for the similarity of some of their stylistic features. For instance, lozenge designs, one of the common features of Gothic chalices, appear on all three chalices analyzed (see detailed descriptions in SI). Such designs also featured on late medieval chalices made across continental Europe, for example, in Germany, the Low Countries and Poland, and were present on pre-Reformation English chalices. In Ireland, the lozenge motif continued well into the early modern period where, despite the arrival of a new Baroque style, many Irish silversmiths continued to employ conservative medieval styles in liturgical ware [1]. Such is the case of the TP-IEP chalice, dated 1589 but still executed in Gothic style. Similar to lozenges, the lancet window design was another international motif, featuring on liturgical objects created in Ireland and elsewhere in medieval Europe and observed in the Ó Learghusa’s knop and in the de Burgo-O’Malley’s stem

### XRF analysis of the chalices

#### Ó Learghusa chalice

**Fig. 2:**
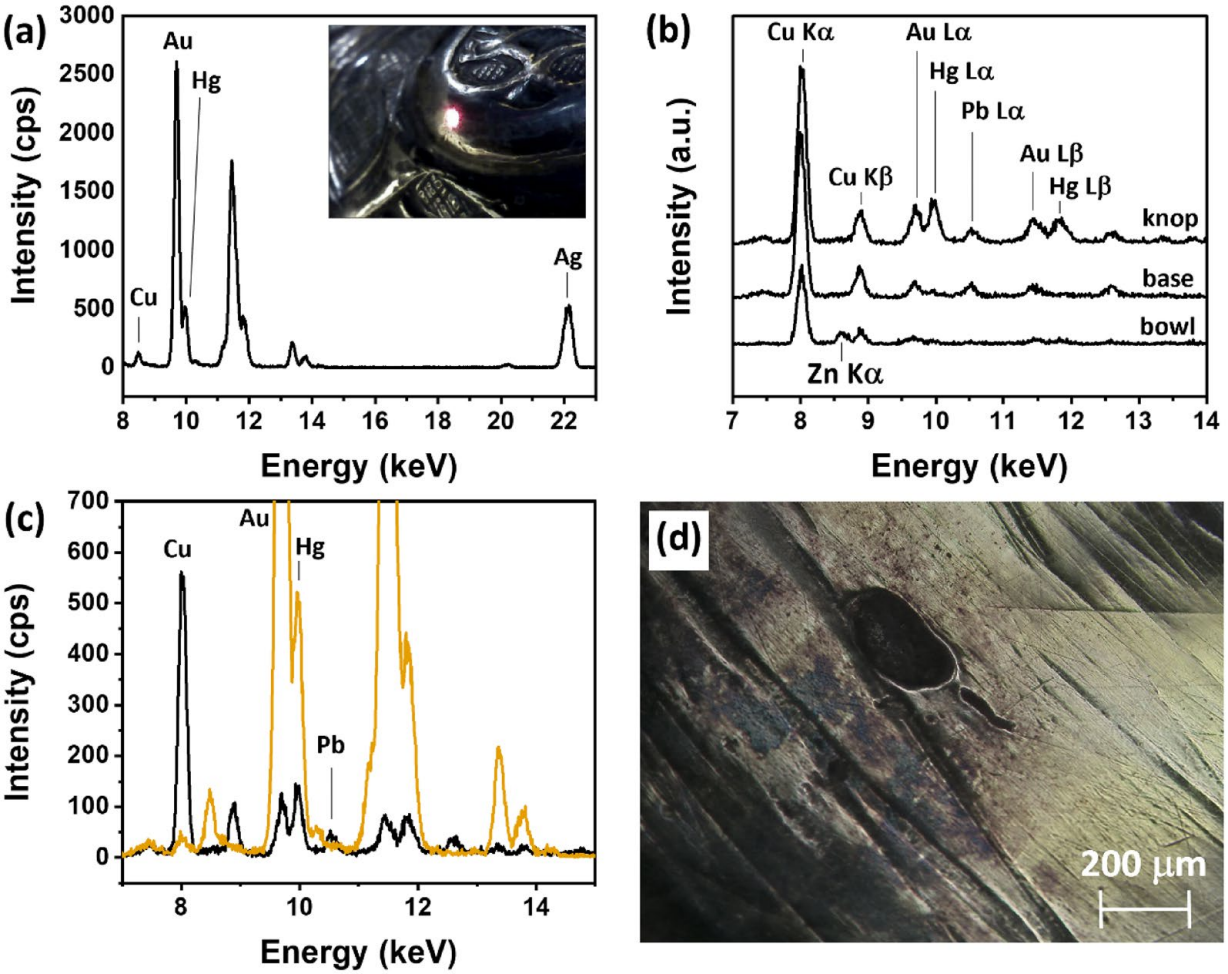
**a** Representative XRF spectrum of gilded areas (taken on the knop) of the Ó Learghusa chalice; **b** Representative XRF spectrum of non-gilded areas of the knop, the base, and the bowl of the chalice; **c** Comparison of XRF spectra from gilded (orange line) and non-gilded (black line) areas. Au peaks have been cut for highlighting trace elements in the region of interest; **d** Photographic detail of the deterioration of the gilding layer

The elemental composition of the Ó Learghusa chalice gilding layer was found consistent across the three parts of the chalice (base, knop and bowl), see Additional file 1: Fig. S1. The representative spectrum showed in Fig. 2a, taken on the gilding layer of the chalice’s knop, was characterized by an intense peak for Au and a relatively intense peak for Hg, confirming the amalgam method of gilding [19]. The presence of Ag and Cu in all the spectra of areas where the base alloy was visibly exposed, defined for simplicity as ‘non-gilded’ (detail in Fig. 2b, full spectra in Additional file 1: Fig. S2), suggested the presence of an Ag/Cu binary alloy, composing the body of the chalice. Despite the inability to quantify the XRF spectra due to lack of standard materials to compare with, the interpretation of the peak areas provided valuable and interesting information. The comparison of XRF spectra taken on gilded and non-gilded areas of the chalice’s base, knop, and bowl, showed that the peak intensity of Au was inversely proportional to that of Ag and Cu (see representative spectra in Additional file 1: Fig. S3). This observation confirmed that Ag and Cu were localized to the subsurface layer, underneath the gilding layer.

Furthermore, a careful assessment of the non-gilded areas in Fig. 2b was carried out. While the knop and the base were characterized by the presence of Cu, Ag, Au, Hg, and Pb, the bowl exhibited Cu, Ag, Au, Hg, and small intensities for zinc (Zn) but no detectable Pb (see also full spectra in Additional file 1: Fig. S2). Ag and Cu together represent the main components of the alloy of the base, the knop, and the bowl. Au and Hg, found at very small intensities in the bowl, are either remnants of the now worn-out gilding layer or detected from adjacent gilded areas. The detection of Pb in the knop and base can be interpreted as an impurity within Ag, as it was never detected in the gilding layer. Pb could either be indicative of Ag extraction through cupellation [11] or could have been added intentionally to improve the workability of Cu [10]. This observation, along with the presence of Zn, may suggest that different raw materials were used for the manufacturing of the bowl compared to the knop and the base. Figure 2c displays a comparison of the XRF spectra of gilded (orange line) and non-gilded areas (black line) of the knop. A significant variation in the peak intensity ratio between Au and Hg was evident. The gilded region predominantly consisted of Au, while non-gilded exhibited a higher peak intensity for Hg compared to Au. This was possibly due to the thinning of the original gilding layer. This finding indicates an enrichment of Hg at the interface of fire-gilded silver substrates, attributed to the process of amalgam/Hg diffusion into the silver substrate [5].

Upon closer microscopic visual examination, it was observed that both the bowl and the base of the chalice exhibited small imperfections on the gilding layer, as depicted in Fig. 2d. These imperfections, taking the form of cavities of varying sizes and shapes, were less present in the knop. Notably, these cavities were observed to be filled with a dark reddish-brown corrosion material possibly ascribable to cuprite (Cu_2_O) [20]. Another form of deterioration, characterized by a dark corrosion layer on the gilding, was also extensively present. This dark layer may indicate the formation of silver and copper sulfides (chalcocite, Cu_2_S; acanthite, Ag_2_S), which deposit on the surface of the gilding layer contributing to its tarnishing [21, 22]. This particular appearance is in line with literature and results from the interaction of environmental gases, primarily hydrogen sulfide (H_2_S) and carbonyl sulfide (COS), with the Ag–Cu alloy. It worth noting that these assertations are not conclusive as based solely on visual assessment and would require further analysis to be confirmed.

#### The de Burgo-O'Malley chalice

**Fig. 3:**
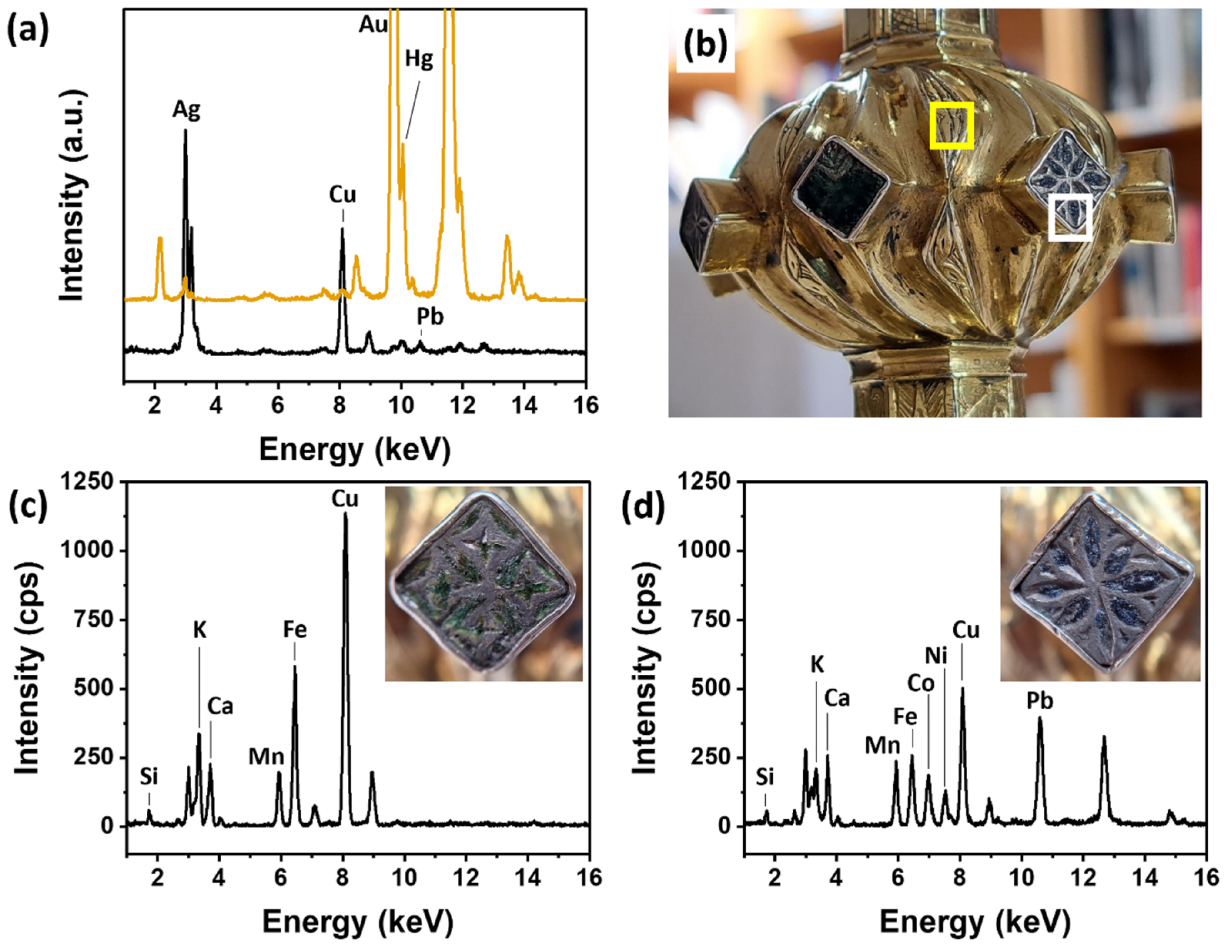
**a** Comparison of the XRF spectra from gilded (orange line) and non-gilded (black line) areas of the de Burgo-O'Malley chalice knop. Au peaks have been cut for highlighting trace elements in the region of interest; **b** Photographic detail of the chalice knop. Boxes indicates where the XRF spectra of a gilded area (yellow box) and non-gilded area (white box) were taken; **c** XRF spectrum of green enamel; **d** XRF spectrum of blue enamel. Insets in **c** and **d** show the analyzed areas

Figure 3 summarizes the elemental characterization of the de Burgo-O'Malley chalice. The elemental composition of both the gilding and base alloy in the base, knop, and bowl of this chalice were similar to each other, suggesting that these components were produced using similar techniques and materials. The XRF spectra of the gilded areas (represented by the orange line in Fig. 3a) confirmed the presence of Au and Hg, indicative of the Hg/amalgam technique utilized for gilding the artifact. Additionally, the presence of Ag and Cu indicated the use of an Ag–Cu alloy for the base (represented by the black line in Fig. 3a), with inclusion of Pb impurities. These were likely originating from the extraction ore or intentionally added to increase Cu workability, similar to the findings in the Ó Learghusa chalice’s knop and base.

The chalice’s enamels underwent elemental investigation. The technique of decorating metals using enamels is a sophisticated craft, and in the case of this silverware, either the *champlevé* or the *basse-taille* [23, 24] technique was used. This was observed through the marks left by the enchasing tool on the metal that remained uncovered after the loss of the enamel layer. The enamels were damaged to the extent that it was not possible to determine if they were originally meant to be transparent or opaque. Figure 3c displays the XRF spectrum of green enamels, revealing the detection of silicon (Si), potassium (K), calcium (Ca), manganese (Mn), iron (Fe), and Cu. Although the Si content is dominant in enamels, representing their matrix made from silicon-based compound, the Si peak intensity was very low as the XRF analysis was performed in air. The high peak intensity for Ag in the background of the analysis area posed a challenge in assessing K, because of the proximity of Ag Lβ line with K Kα line in the spectrum. However, a semi-quantitative approach using PyMca software allowed to detect K despite the interference, and its presence was attributed to the use of potash in the glass production. The presence of Ca, Mn, and Fe, along with K, were suggestive of the use of various fluxing agents to lower the melting point of the siliceous matrix [25, 26]. Cu, detected at higher peak intensities than the Ag–Cu alloy in background (see Additional file 1: Fig. S4), suggested the use of Cu-based pigment for obtaining the green color of the glass. Considering the high peak intensity for Fe, it cannot be excluded that a Fe-based pigment was also use for imparting a green color to the glass [27]. Cu alone may impart a bluish hue, and the addition of Fe compound(s), yellow in color, may serve to shift the overall color towards a greener shade. Figure 3d displays an XRF spectrum taken on blue enamel. Si, K, Ca, Mn, and Fe were ascribed to the ingredients composing the glass matrix. Cobalt (Co) was detected at relatively high peak intensity. It is known that Co-based compounds were used as blue coloring agents in historical glasses [28]. Nickel (Ni) was detected at a similar peak intensity compared to Co. The co-presence of Co and Ni suggests the use of smalt for the blue coloring of this enamel. Studies highlighted that presence of Ni, arsenic (As), bismuth (Bi), and Ag, alone or in combination, may suggest a provenance of the cobalt-based pigment, not investigated in this study [29, 30]. Pb, detected at a relatively high peak intensity, strongly suggested the use of a Pb-based material possibly added as an opacifier and for lowering down the melting point of the flux [28].

#### The TP-IEP chalice

**Fig. 4:**
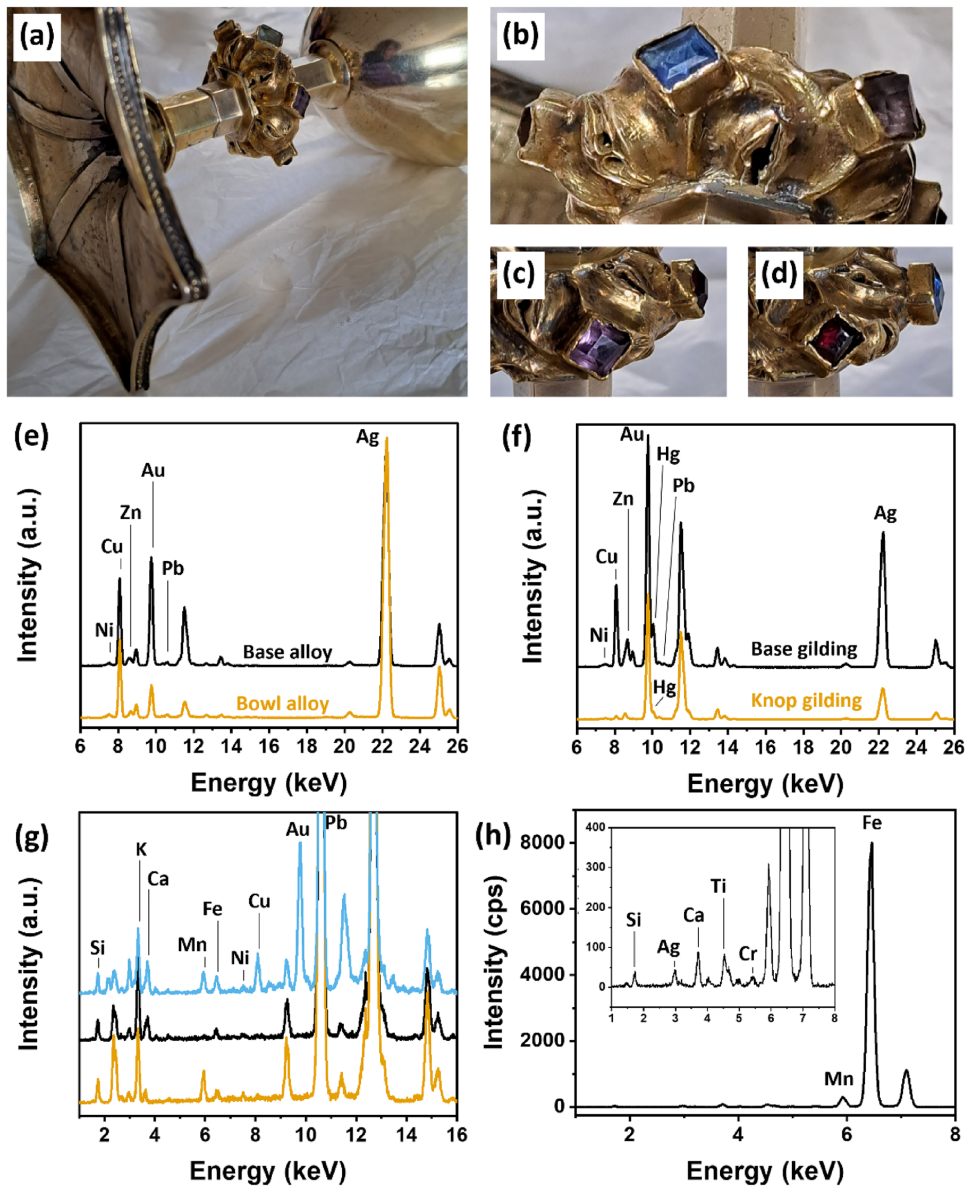
**a** Photographic detail of the TP-IEP chalice, view form below; **b** Detail of the knop with the silver-gray alloy visible, as well as the blue glass; **c** Detail of the purple glass; **d** Detail of the red glass; **e** Comparison of the XRF spectra from the bowl alloy (bottom orange line) and base alloy (top black line). **f** Comparison of the XRF spectra from the knop gilding (bottom orange line) and base gilding (top black line); **g** Comparison of the XRF spectra from the transparent glass (top blue line), blue glass (middle black line) and purple glass (bottom orange line); **h** XRF spectrum of red glass. The inset display a zoom in of the same spectrum to highlight the elements detected at low peak intensities. Pb and Fe peaks in **g** and **h** were cut for highlighting traces elements in the region of interest

During visual assessment of the TP-IEP chalice, it was noticed that the typical porous surface texture commonly associated with fire-gilding, caused by the evaporation of Hg [10], was absent on the bowl and the base. Additionally, the bottom of the base appeared to have a golden color (see Fig. 4a). This was intriguing, as areas usually hidden from view were typically not embellished with precious and expensive gold leaf. This observation led to the suggestion that a different manufacturing technique or alloy might have been used for these parts of the TP-IEP chalice, setting these apart from the Ó Learghusa and the de Burgo-O’Malley chalices. In contrast, the TP-IEP knop’s alloy appeared silver-gray with evidence of a gilding layer (as visible in the photographic detail of Fig. 4b), suggesting that Au was applied on top of the surface. These findings supported the theory that the chalice had its base and bowl refurbished, possibly along with the colored decorative glass mounted in the lozenges of the knop (as observed in Fig. 4a–d).

The elemental analysis of the base and the bowl (Fig. 4e) revealed the presence of Ni, Cu, Zn, Au, Pb, and Ag. While XRF analysis in metal studies does not directly assess bulk composition but rather surface characteristics, the presence of a ternary alloy is a possibility. It is hypothesized that the TP-IEP chalice's bowl and base could be crafted from a ternary Ag–Cu–Au alloy with impurities of Ni, Zn, and Pb, considering factors such as the absence of Hg in the spectra, which would indicate the presence of a fire-gilded layer, the uniform surface texture observed, the color of the metal, and the peak intensity ratio of these elements. Further analysis would be required to confirm this possibility.

Interestingly, the upper part of the base of the TP-IEP chalice was also fire-gilded, possibly in an attempt to uniform its color to the color of the knop. Figure 4f displays the XRF spectra of the gilding of the upper part of the base and the knop. It is visible that both spectra show the presence of Au and Hg, suggesting the detection of Hg/amalgam in both areas. However, the peak intensity ratios of Au vs Hg in these two parts of the chalice were found to be different, suggesting a different manufacturing process. In addition, the gilding of the knop did not contain traces of Ni, Zn, and Pb, as observed in the alloy of the base and bowl. Unfortunately, it was not possible to access areas of the knop without gilding, therefore the composition of its base alloy is still unknown.

The decorative transparent glass, along with the blue and the violet glasses (represented by the blue, black, and orange line in Fig. 4g, respectively), were characterized by the presence of Pb, Si, K, Ca, Mn, Fe, and Ni. Pb, Si, and K suggest the general composition of lead glasses, which were not produced before 1674 [31], composed mainly by silica, lead oxide, and potash. This reinforce the hypothesis that the decorative glasses of the TP-IEP chalice’s knop may not be contemporary with the production of the knop itself (1589). The presence of Ni, found with a comparable peak intensity in all three samples, was considered a trace element of potash within the glass matrix [32]. Ca was attributed to the use of CaO as a stabilizing component of the flux [33]. Fe was found with small peak intensity in all samples and was ascribed to the presence of Fe-based compounds possibly used as coloring agents. The main difference among the samples lied in the content of Mn, which showed small peak intensity in both the transparent and purple glass and was absent in the blue glass. It is known that Mn was used to achieve different effects, serving as a decoloring agent in small amounts or providing a purplish color when used in higher quantities [34]. Ag, Cu, and Au in transparent glass were interpreted as interference from the surrounding area (gilded Ag–Cu alloy). In Fig. 4h, the spectrum captured from the red glass reveals predominant peak intensities for Fe and Mn, with Si, Ca, titanium (Ti), and chromium (Cr) registering at very low peak intensities (refer to the inset in Fig. 4h). While Si, and Ca are possibly attributed to the glass matrix, and Ag possibly arising from the metal alloy in the background, the compounds responsible for the distinct red coloration of the glass remain a subject of ongoing debate.

## Conclusions

This study reveals the outcomes of an XRF investigation into three significant late medieval chalices associated with Ireland. Both the Ó Learghusa and de Burgo-O'Malley chalices showcase craftsmanship from an Ag/Cu alloy, embellished with the fire-gilding technique. The analysis delves into the blue and green enamels on the de Burgo-O'Malley, elucidating their composition based on Co and Fe/Cu glass, respectively. In contrast, the TP-IEP chalice boasts a more intricate construction, featuring the fire-gilding of the Ag/Cu alloy of the knop and possibly a ternary alloy composition (Ag/Au/Cu) for the base and the bowl. This complexity suggests that these components may not align chronologically with the production of the chalice knop. Further scrutiny of the TP-IEP chalice's transparent, blue, and purple glass unveils the identification of lead-potash glass, a type not manufactured before 1674. This pivotal finding substantiates the hypothesis that the decorative glasses on the TP-IEP chalice's knop may not coincide with the knop's production in 1589. The distinct composition aligns more closely with lead glasses produced after 1674, indicating a potential later addition or alteration to the chalice. The analysis of the red glass reveals a notable presence of Fe, with minor peak intensities for Ti and Cr. This additional insight adds to the complexity of the chalice's composition. Further investigation into the historical context and production methods of these glasses may provide additional insights into the timeline and origin of the decorative elements on the chalice. In conclusion, these groundbreaking findings shed light on the diverse manufacturing methods employed in creating historically significant chalices, offering valuable insights into the artistic craftsmanship of medieval Ireland.

### Supplementary Information

**Additional file 1: Figure S1.** Comparison of gilded areas of the bowl (black spectrum), knop (blue spectrum), and base (orange spectrum) of the Ó Learghusa chalice. **Figure S2.** Comparison of non-gilded areas of the base (black spectrum), knop (blue spectrum), and bowl (orange spectrum) of the Ó Learghusa chalice. **Figure S3.** Representative XRF spectra showing comparison between non-gilded area (black spectrum) and gilded area (orange spectrum) of the Ó Learghusa chalice’s base. **Figure S4.** Comparison of non-gilded area of the knop (black spectrum) and green enamel (orange spectrum) of the Burgo-O'Malley chalice.

## Data Availability

The data that support the findings of this study are available from the corresponding author, D.I., upon reasonable request.
