# Supplementary material for: X-ray fluorescence analysis of three late medieval silver chalices associated with Ireland
Source: Herit Sci. 2024 Apr 25;12(1):130. doi: 10.1186/s40494-024-01240-2 (PMC11045461; doi:10.1186/s40494-024-01240-2)
Supplement: Supplementary file 1 — Additional file 1: Figure S1. Comparison of gilded areas of the bowl (black spectrum), knop (blue spectrum), and base (orange spectrum) of the Ó Learghusa chalice. Figure S2. Comparison of non-gilded areas of the base (black spectrum), knop (blue spectrum), and bowl (orange spectrum) of the Ó Learghusa chalice. Figure S3. Representative XRF spectra showing comparison between non-gilded area (black spectrum) and gilded area (orange spectrum) of the Ó Learghusa chalice’s base. Figure S4. Comparison of non-gilded area of the knop (black spectrum) and green enamel (orange spectrum) of the Burgo-O'Malley chalice. [file 40494_2024_1240_MOESM1_ESM.docx]

**Additional Material**

**Description of the chalices.** The Ó Learghusa chalice is relatively small, standing at 16cm in height, with its bowl being 10.3cm in diameter and its foot 12.2cm in diameter. The stem with the knop is the most ornate part of the chalice. Around the middle part of the knop there are twelve lozenges (rhombus shapes) filled with plant motifs, which intersect to form the x shape; one of the lozenges bears the sign of the cross. Above and below the lozenges there are twelve lobes (six on either side of lozenges), each decorated with a leaf design. Between the lobes there are designs that look like elongated Gothic lancet windows. The foot of the chalice is circular with a flattened rim: three small holes and a rivet suggest that the foot bore a figure of Christ crucified or the cross, a typical representation for the liturgical plate at that time. The absence of any inscriptions or hallmarks on the chalice posed a challenge in assigning a specific provenance or date to the artifact. The foot is circular, where typically a polygonal shape (hexagonal or octagonal) is a feature in chalices dating from the later medieval period. These non-typical shapes of the bowl and the foot suggest that the Ó Learghusa chalice may be a composite object.

The de Burgo-O’Malley chalice measures 20.3cm in height, its wide bowl is 10.5cm in diameter and its foot is 16.2cm wide. The chalice has an octagonal stem with chased decoration and a knop with eight projecting lozenges, each with translucent enamel. The stem is engraved with different designs: four undecorated walls of the stem above the knop alternate with four panels decorated with lancet window designs; below the knop four stem panels display lancet window designs that alternate with plant motifs. The foot is octagonal with incurved angle and base lines. The foot is engraved with the sacred monogram (IHC) and the Latin inscription that reads “Thomas de Burgo et Grania Ni Malle me fieri fecerunt Anno Domini MCCCCLXXXXIIII”.

The TP-IEP chalice measures 15cm in height, the bowl is 9.6cm in diameter and the foot is 11.9cm wide. The bowl of the chalice is wide and splayed. A plain stem is hexagonal in section with a flattened knop. The knop is embellished with openwork designs and six projecting lozenges, each filled with color glass. The foot is a flattened hexagonal pyramidoid with incurved angle and base lines. It is inscribed with the words: ‘TP ET IEP ME FIERI FECERVT QVORVM ANIMABVS MISERIATVR OMNIPOTENS 1589’. One facet of the foot bears the image of the Paschal Lamb with a cross. The gilding on the bowl and on portions of the stem appear paler than the knop and the foot suggesting that these parts may be later replacements.


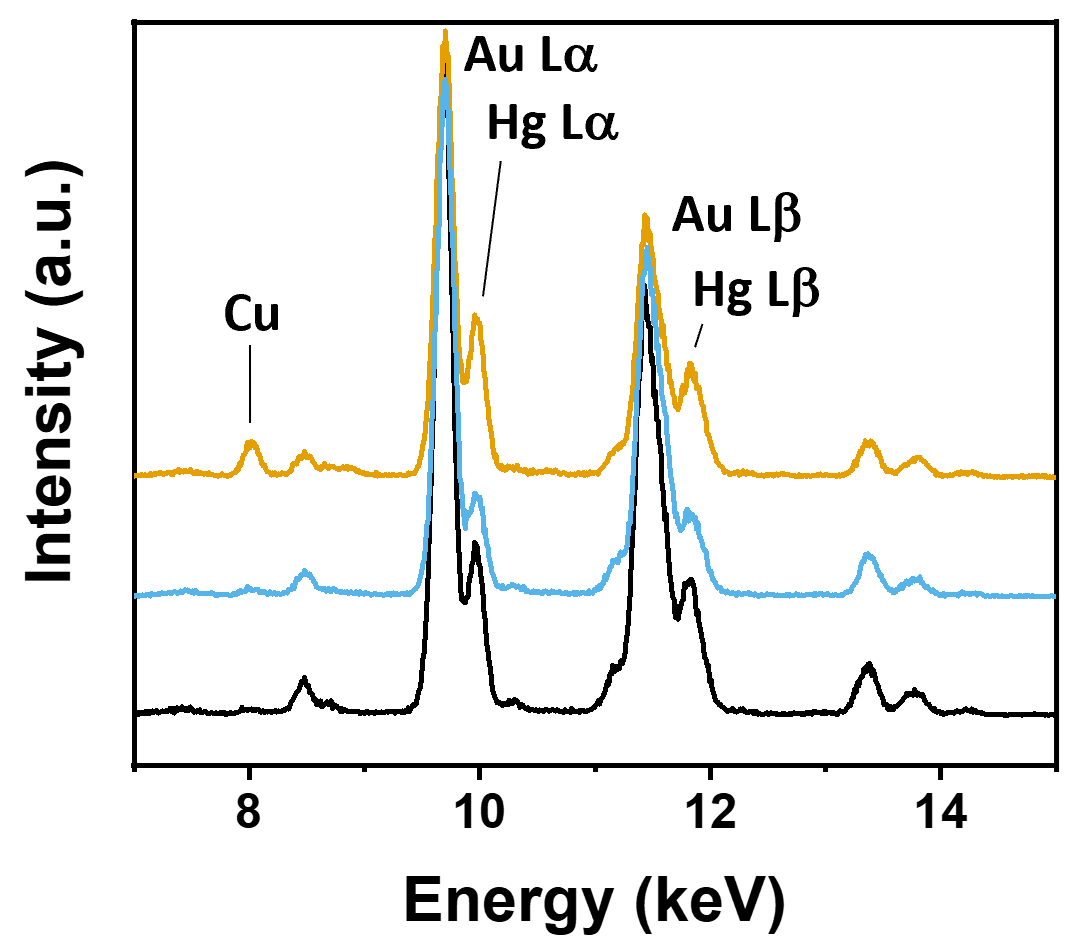


**Figure S1.** Comparison of gilded areas of the bowl (black spectrum), knop (blue spectrum), and base (orange spectrum) of the Ó Learghusa chalice.


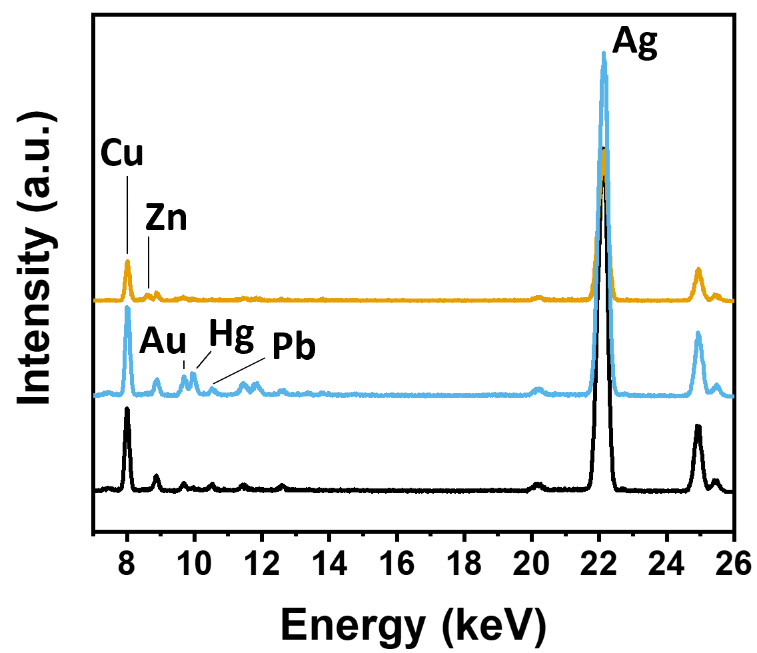


**Figure S2.** Comparison of non-gilded areas of the base (black spectrum), knop (blue spectrum), and bowl (orange spectrum) of the Ó Learghusa chalice.


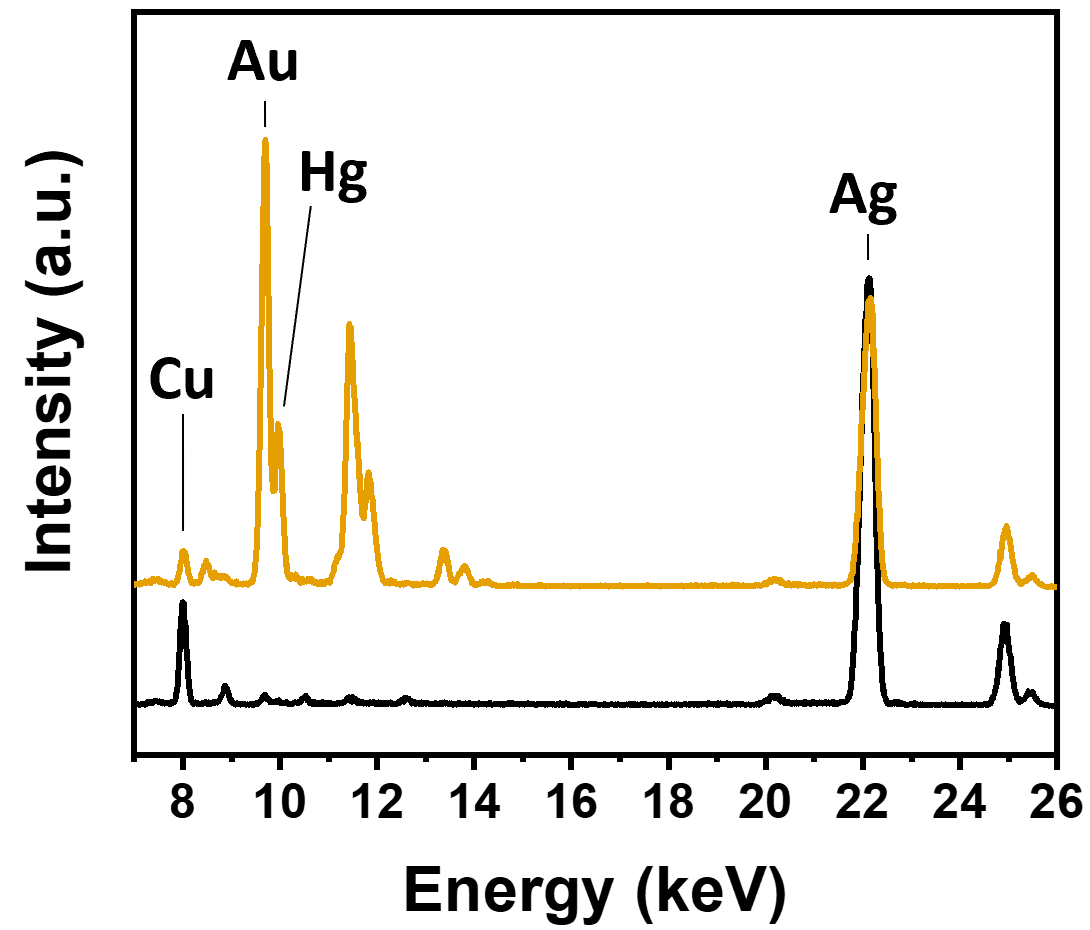


**Figure S3.** Representative XRF spectra showing comparison between non-gilded area (black spectrum) and gilded area (orange spectrum) of the Ó Learghusa chalice’s base.


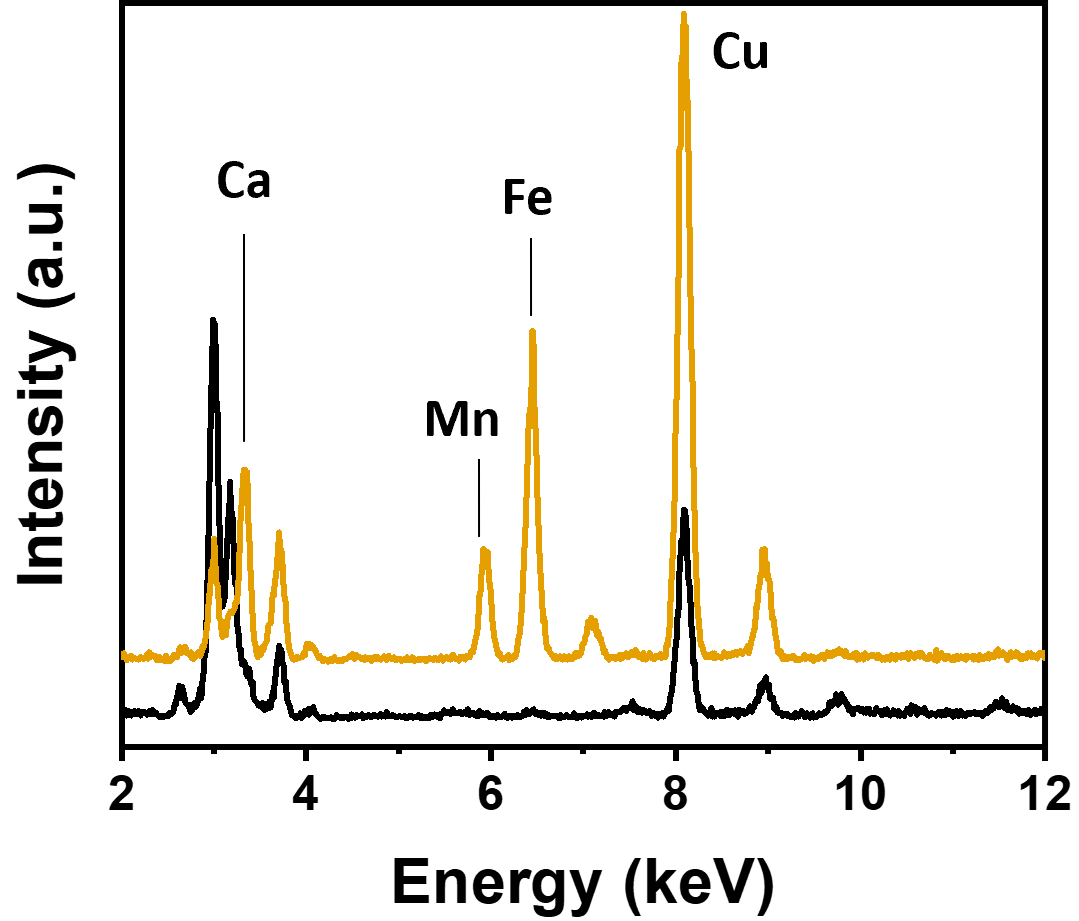


**Figure S4.** Comparison of non-gilded area of the knop (black spectrum) and green enamel (orange spectrum) of the Burgo-O'Malley chalice.
